# Genetic testing and Guangdong college students in China: A cross-sectional study of knowledge and attitudes

**DOI:** 10.1016/j.pmedr.2025.103133

**Published:** 2025-06-08

**Authors:** Jiaming Wang, Ruoru Lin, Sijing Luo, Beilei Zhong, Yurang Zhu, Jiayi Huang, Dangui Zhang, William Ba-Thein

**Affiliations:** a Undergraduate Research Training Program (UGRTP), Shantou University Medical College, Shantou, People's Republic of China; b Department of Dermatology, Peking University Shenzhen Hospital, Shenzhen, People's Republic of China; c School of Stomatology, Jinan University, Guangzhou, People's Republic of China; d Department of Cardiovascular Medicine, Third Affiliated Hospital of Sun Yat-sen University, Guangzhou, People's Republic of China; e Zhejiang University School of Medicine, Hangzhou, People's Republic of China; f Department of Gynecology and Obstetrics, The First Affiliated Hospital of Jinan University, Guangzhou, People's Republic of China; g Southern Medical University, Guangzhou, People's Republic of China; h Research Center of Translational Medicine, Second Affiliated Hospital of Shantou University Medical College, Shantou, People's Republic of China; i Clinical Research Unit, Shantou University Medical College, Shantou, People's Republic of China; j Department of Microbiology and Immunology, Shantou University Medical College, Shantou, People's Republic of China

**Keywords:** Direct-to-consumer genetic tests, Informed consent, Functional health literacy, College students, China

## Abstract

**Objective:**

This study investigated functional health literacy and attitudes toward genetic testing among Chinese college students to understand if they can make informed decisions and avoid potential risks.

**Methods:**

A cross-sectional anonymous online survey was conducted with students from 13 colleges in Guangdong province during October–November 2021.

**Results:**

Of the 1543 participants, 53.9 % were female, predominantly undergraduates (97.2 %). Most students reported being health-conscious (76.6 %), less religious (60.3 %), possessing health esteem (76.4 %), and having parents in non-healthcare occupations (89.1 %). The median score for functional genetic literacy was 10 out of 17. Most (91.4 %) expressed positive attitudes toward genetic testing, with 65.1 % favoring direct-to-consumer options. The most preferred tests were predictive (83.3 %) and premarital/preconception tests (76.4 %). Multiple linear regression analysis showed independent predictors of functional genetic literacy as female gender (Beta, 95 %CI: 0.26, 0.02–0.49), medicine major (1.51, 1.27–1.74), health-esteem personality trait (0.34, 0.06–0.62), lower religiosity (0.66, 0.49–0.83), and lower perceived financial status (0.47, 0.21–0.73). Logistic regression analysis identified independent predictors of positive attitude toward genetic testing as self-respect personality (adjusted odds ratio, 95 %CI: 1.81, 1.23–2.66), lower perceived financial status (0.57, 0.38–0.83), and parental occupation in non-healthcare (0.38, 0.17–0.89).

**Conclusions:**

Guangdong college students were mostly positive toward genetic testing, particularly predictive and premarital options. However, their limited functional genetic testing literacy may hinder informed decision-making. To address ethical, legal, and social implications, China should strengthen public education, promote the genetic counseling profession and enhance regulatory oversight in the genetic testing industry.

## Highlights

• Functional genetic testing literacy assessment with students of Guangdong colleges.
• High positive attitudes despite limited functional genetic literacy among students.
• Predictive and premarital/preconception tests as their favorites.
• Gender, major, personality, religiosity, and finance influencing genetic literacy.

## 1 Introduction

Genetic testing refers to the analysis of an individual's chromosomes, genes, or gene products for any defects or variations (Mirae Asset Asia Pacific Research, 2020). Genetic testing is categorized as predictive genetic testing (predicting any increased risk for a particular disease in a pre-symptomatic individual), genetic screening (screening for a genetic disease in an asymptomatic individual at preconception, prenatal, or postnatal periods), diagnostic genetic testing (identifying a genetic disease in a symptomatic individual), and pharmacogenomics (studying individual's genes for personalized medication) and tumor analysis (investigating genetic markers in a patient for suitable treatment options) (Mirae Asset Asia Pacific Research, 2020; Katsanis and Katsanis, 2013).

Along with recent advancements in big data, machine learning, and artificial intelligence, genetic testing is revolutionizing precision public health by enhancing surveillance, risk assessment, and disease detection with tailored interventions. Genetic testing can provide valuable information about an individual's health status or potential risk of certain genetic conditions, thereby allowing personal empowerment to make informed decisions on healthcare management, disease prognosis, and family planning (Katsanis and Katsanis, 2013). However, there are also risks associated with genetic testing, such as adverse consequences from uncertain results, secondary findings, psychological impacts on individuals and families, compromised family values, privacy concerns, and social stigma (Mayo Clinic, 2020; Katsanis and Katsanis, 2013; Zhong et al., 2021).

According to Grand View Research, the global genetic testing market size valued at USD 7421 million in 2022 is projected to grow at a compound annual growth rate of 22 % from 2023 to 2030 (Grand View Research, 2022). China also saw exponential growth in its direct-to-consumer (DTC) genetic testing industry (Du and Wang, 2020; Zhu, 2022), with 12.1 million consumers in 2020 (Zhu, 2022) and a market size of USD 4.9 billion in 2024 as reported by the International Market Analysis Research and Consulting Group (IMARC, 2023). The rapid expansion of the global genetic testing market raises significant concerns regarding its ethical, legal, and social implications for medicine, public health, and social policy.

From the public health perspective, individuals considering genetic testing should be aware of the complexity inherent in genetic testing and be fully informed before making decisions. However, studies have shown that global citizens including well-educated people have inadequate knowledge of genetic testing (Chapman et al., 2019; Ganne et al., 2021). The population of our research interest was Chinese college students because they represent potential consumers of genetic testing. To assess their interest in genetic testing and their ability to make informed decisions, we investigated their functional genetic literacy (the ability to understand the benefits, risks, and limitations of genetic testing for making informed choices about genetic health risks and family planning) and their attitudes toward genetic testing.

## 2 Methods

### 2.1 Study design, participants, and study site

This study was a cross-sectional self-administered anonymized online survey conducted with students from colleges and universities in Guangdong province, China, following the Consensus-Based Checklist for Reporting of Survey Studies guideline (Sharma et al., 2021). In general, after completing 12 years of primary and secondary education, students enter colleges or universities at the age of 17–18 years. The distinction between “college” and “university” is minimal, both offering tertiary education. Bachelor's programs typically last four years (longer for majors like medicine), master's programs two to three years, and doctoral programs three to four years.

### 2.2 Ethics approval

This study was approved by the Ethics Committee of Shantou University Medical College (SUMC-2021-34). The study met the institution's guidelines for protection of human subjects concerning safety and privacy. Study participants provided informed consent by agreeing to the study objectives, content, and data usage before participating in the survey.

### 2.3 Survey instrument development

We used a survey instrument with 50 question items to collect information on participant characteristics (eleven items), awareness of genetic testing (four items), genetic literacy (ten items), risks and limitations of genetic testing (seven items), attitude toward genetic testing (fifteen items), and opinions about genetic testing (three items) (Supplementary File 1).

Functional genetic literacy in the context of genetic testing was defined herein as `the ability to understand fundamental genetic concepts, the benefits, risks, and limitations of genetic testing, and the interpretation of test results to make informed decisions about genetic health risks and family planning`. The questions related to functional genetic literacy (Supplementary File 2) were designed based on a previous study (Fitzgerald-Butt et al., 2016). Due to the lack of a suitable validated instrument to evaluate the constructs of our interest, we self-developed and validated the questionnaire.

#### 2.3.1 Content validity

Two senior investigators validated the survey content to cover all aspects relevant to our research question.

#### 2.3.2 Translation validity

A forward-backward translation method was applied to translate our questionnaire. Two bilingual investigators independently translated the original English questionnaire into Chinese, and two others back-translated it into English to identify and correct any inconsistencies or shifts in meaning. Translating different genetic testing methods was based on the Chinese terms that appeared in the top three formally registered companies in China, including Beijing Genomic Institutes, Berry Genomics (Beijing Berry Genomics Co., Ltd.), and Novogene (Beijing Novogene Technology Co., Ltd.).

#### 2.3.3 Face validity

We had a pretest with a group of volunteers (10 college students) and requested their feedback on the clarity, understanding, interpretation, answerability, response latency (time required to answer), and cultural appropriateness.

#### 2.3.4 Pilot testing

We recruited 30 college student volunteers from different universities in Guangdong to ensure the functionality of the whole survey process. The questionnaire was revised based on their feedback.

#### 2.3.5 Survey administration

Posters and leaflets with a quick-response code were distributed in universities within Guangdong province and shared on WeChat for voluntary participation through the Chinese survey hosting site Sojump (www.wjx.cn) during October – November 2021. A small monetary incentive (One RMB = 0.14 USD) was provided to encourage participation. Only one submission per Internet Protocol address was allowed. We only enrolled respondents from colleges and universities in Guangdong Province by verifying their Internet Protocol addresses and locations.

### 2.4 Data management and analysis

Survey data were imported from the survey website to an Excel database. Multiple submissions, inconsistencies, and incomplete responses were removed. We analyzed only completed questionnaires. Participating colleges (*n* = 13) were categorized into medical and non-medical for subgroup analysis. Non-normally distributed continuous variables, such as functional genetic knowledge, were analyzed using the Mann-Whitney *U* test or Kruskal-Wallis H test. Categorical variables including sex, major, program, hometown, personality, religiosity, parental occupation, and perceived family financial status were analyzed using the Chi-square test. Demographic factors associated with positive attitudes toward genetic testing or genetic testing literacy were analyzed using multiple logistic regression or multiple linear regression, respectively.

Based on the median (interquartile range, IQR) of the observed knowledge scores, functional genetic literacy was classified as low (below Q1, ≤ 7), moderate (between Q1 and Q3, 8–12), and high (Q3 and above, ≥ 12).

Positive attitude toward genetic testing was defined based on the “YES” answer regarding the first question of attitude toward genetic testing section: Would you take predictive genetic tests to know if you are at risk of developing diseases?

All statistical tests were two-tailed, and statistical significance was set at a *P*-value of <0.05. SPSS version 21.0 was used for data analysis.

## 3 Results

### 3.1 Participant characteristics (Table 1)

During one month, the survey site received 1607 visits, and 1543 (96.0 %) were eligible for the study. Respondents represented 13 colleges/universities in Guangdong province. Most participants were young (median age, IQR: 20, 19–22; range, 16–40), females (53.9 %, 832/1543), undergraduates (97.2 %, 1500/1543), and Guangdong natives (61.8 %, 953/1543), with parents in non-healthcare occupation (89.1 %, 1375/1543), and perceived family income as average (77.9 %, 1202/1543). Non-medicine majors accounted for 52.6 % (811/1543). Most students considered themselves as health-conscious (76.6 %, 1182/1543), having health esteem (76.4 %, 1179/1543), or possessing self-respect (48.0 %, 741/1543). Many (60.3 %, 931/1543) reported being either not religious or only a little religious.

**Table 1:** Functional genetic knowledge and attitude toward genetic testing among college students in Guangdong, China (2021).

	Total *N* = 1543 (%)	Functional genetic knowledge (max. 17) 10 (7, 11)^1^	Positive attitude toward genetic testing^2^ 1410 (91.4)
Gender		*P* < 0.05	*P* = 0.16
Female	832 (53.9)	10 (7, 12)	768 (92.3)
Male	711 (46.1)	9 (7, 11)	642 (90.3)
Age group^3^		*P* < 0.01	*P* = 0.22
16–24	1345 (87.2)	10 (8, 12)	1223 (86.7)
25–30	132 (8.5)	7 (7, 8)	123 (93.2)
31–35	48 (3.1)	7 (7, 8)	37 (97.4)
36–40	18 (1.2)	7 (7, 8)	27 (96.4)
Program		*P* = 0.18	*P* = 0.31
Undergraduate	1500 (97.2)	10 (7, 12)	1369 (91.3)
Graduate	43 (2.8)	9 (7, 11)	41 (95.3)
College		*P* < 0.01	*P* = 0. 60
Non-Medicine	811 (52.6)	8 (7, 10)	744 (91.7)
Medicine	732 (47.4)	11 (9, 12)	666 (91.0)
Hometown		*P* < 0.01	*P* = 0.60
Guangdong	953 (61.8)	11 (8, 12)	868 (91.1)
Non-Guangdong	590 (38.2)	8 (7, 10)	542 (91.9)
Self-reported personality trait (multiple choice question)^4^			
Health-conscious	1182 (76.6)	10 (7, 12) 5	1088 (92.0)
Health esteem	1179 (76.4)	10 (7, 12)^6^	1085 (92.0)
Self-respect	741 (48.0)	9 (7, 11) 7	696 (93.9)^8^
Others 9	1180 (76.5)	10 (7, 12)	1083 (91.8)
Perceived religiosity		*P* < 0.01	*P* = 0.13
Very much-much	201 (13.0)	7 (7, 9)	191 (95.0)
Neutral	411 (26.6)	10 (7, 11)	371 (90.3)
Little-not at all	931 (60.4)	10 (8, 12)	848 (91.1)
Parental occupation		*P* = 0.20	*P* = 0.01
Non-healthcare	1375 (89.1)	10 (7, 12)	1248 (90.8)
Healthcare	168 (10.9)	9 (7, 12)	162 (96.4)
Perceived family financial status		*P* < 0.01	*P* < 0.01
Very good-good	160 (10.4)	7 (7, 9)	153 (95.6)
Average	1202 (77.9)	10 (7, 12)	1103 (91.8)
Poor–very poor	181 (11.7)	11 (8, 12)	154 (85.1)

1 Functional genetic knowledge = the ability to understand fundamental genetic concepts, the benefits, risks, and limitations of genetic testing, and the interpretation of test results to make informed decisions about genetic health risks and family planning. Data shown as median (IQR) and analyzed by the Mann-Whitney U test or Kruskal-Wallis H test.

2 Positive attitude toward genetic testing was defined based on the “YES” answer to “Would you take predictive genetic tests to know if you are at risk of developing diseases?” Data shown as n (%) and analyzed by the Chi-square test.

3 including younger accelerated undergraduate students and mid-career graduate students.

4 self-perceived responses.

5 *P* < 0.01.

6 *P* < 0.01*.*

7 *P* = 0.01*.*

8 *P* < 0.01*;* all *P*-values compare respondents who reported a given personality trait against who did not.

9 including perfectionist, worrisome, depression-prone, shy, and outgoing personalities. All *P*-values compare respondents who reported a given trait against those who did not (via multiple-checkbox responses).

### 3.2 Functional genetic literacy (Table 1 and Supplementary File 3)

Students had a moderate level of functional genetic literacy (median IQR: 10, 7–11; equivalent to 58.8 %, 41.2 % ∼ 64.7 % of the total score). Significantly higher scores were observed with females (*P* < 0.05, vs. males), younger age group (16–24 years old) (*P* < 0.01, vs. > 24 years old), medical students (*P* < 0.01, vs. non-medical students), and Guangdong natives (*P* < 0.01, vs. non-Guangdong natives).

### 3.3 Participants’ awareness of genetic testing and its benefits (Supplementary File 4)

Participants exhibited high awareness of premarital genetic testing (91.8 %), followed by preconception testing (90.9 %) and prenatal testing (88.0 %). Regarding the perceived benefits of genetic testing, participants recognized its value in predicting risk (90.4 %), identifying the cause of genetic conditions (70.3 %), understanding one's own genes (82.5 %), and guiding fertility decisions (73.5 %).

### 3.4 Attitudes and opinions toward genetic testing and testing companies (Supplementary Files 3 & 4)

Most participants (91.4 %) expressed positive attitudes toward undergoing one or more genetic tests, with many expressing their willingness to take DTC home genetic testing (65.1 %) or to get tested even for unpreventable or untreatable disorders (61.6 %).

Regarding trust in genetic testing providers, participants considered domestic genetic testing companies the most trustworthy (73.8 %), followed by foreign companies (21.7 %) and online testing services (2.8 %). When asked, *“Do you trust genetic testing companies in protecting your privacy and not misusing your genetic data?”* most students responded *“yes”* (73.2 %). Additionally, they viewed genetic testing as a personal responsibility to maintain their own health and pass down healthy genes to future generations (89.5 %) (Supplementary File 4).

### 3.5 Preferred genetic tests (Fig. 1)

Predictive and premarital/preconception genetic tests were the top category choices among the participants (83.3 %, 1286/1543; and 76.4 %, 1179/1543, respectively). The most preferred choices among the available genetic tests within each category were whole genome sequencing in the predictive testing (51.0 %, 656/1286), detection of monogenetic hereditary diseases in the premarital/preconception testing (64.6 %, 756/1179), detection of noninvasive fetal chromosomal abnormality in the prenatal testing (65.0 %, 644/991), and newborn genetic screening in the neonatal testing (64.8 %, 589/909).

**Fig. 1:**
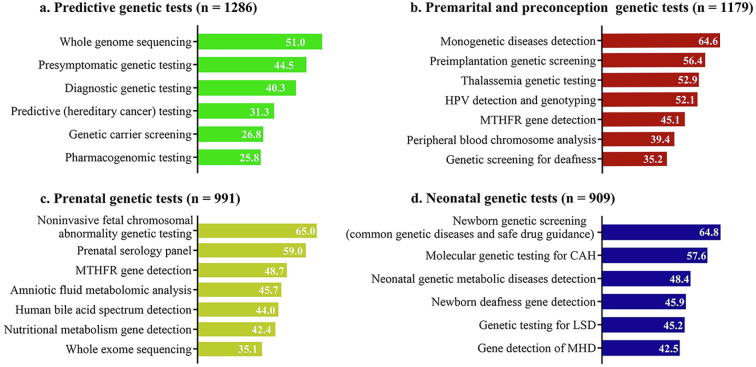
Preferred genetic tests among college students in Guangdong, China (2021). Note: Values represent percentages from multiple responses. Analysis restricted to respondents who indicated willingness to take one or more of the following tests: predictive genetic testing, premarital/preconception genetic testing, prenatal genetic testing, or neonatal genetic testing; Abbreviations: HPV, human papillomavirus; MTHFR, methylenetetrahydrofolate reductase; CAH, congenital adrenal hyperplasia; LSD, lysosomal storage disease; MHD, metabolic hereditary disorder.

### 3.6 Factors associated with functional genetic literacy and positive attitudes toward genetic testing (Table 1 and Fig. 2)

In univariate analyses, the functional genetic literacy score was significantly associated with religiosity and perceived family financial status (*Ps* < 0.01). The association was also significant between the overall positive attitude and self-respect personality (*P* < 0.01), parental occupation (*P* = 0.01), and perceived family financial status (*P* < 0.01). A significant correlation was observed between genetic testing literacy and attitudes (*r*_*rb*_  = 0.05, *P* = 0.03; by the Mann-Whitney *U* test) but with identical median scores and overlapping IQRs between positive- and negative-attitude groups, i.e., 10 (9–12) vs. 10 (7–12), indicating no meaningful relationship between them.

**Fig. 2:**
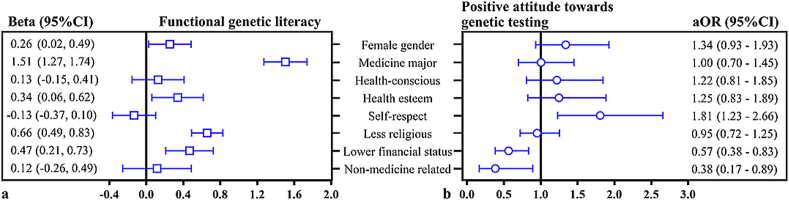
Factors associated with functional genetic literacy and positive attitude toward genetic testing among college students in Guangdong, China (2021); Note: (A) Multiple linear regression; (B) Multiple logistic regression. Functional genetic knowledge = the ability to understand fundamental genetic concepts, the benefits, risks, and limitations of genetic testing, and the interpretation of test results to make informed decisions about genetic health risks and family planning. Positive attitude toward genetic testing was defined based on the “YES” answer to “Would you take predictive genetic tests to know if you are at risk of developing diseases?” Adjusted for age, academic program, and hometown. Personality traits (health consciousness, health esteem, self-respect) and religiosity were self-reported. “Non-medical related” refers to parental occupations, not respondents.

Multiple linear regression analyses showed independent relationships between functional genetic literacy and female gender (Beta, 95 %CI: 0.26, 0.02–0.49), medicine major (1.51, 1.27–1.74), having health esteem (0.34, 0.06–0.62), being less religious (0.66, 0.49–0.83), or lower perceived financial status (0.47, 0.21–0.73).

Also, independent relationships were observed between students' attitudes toward genetic testing and self-respect personality (adjusted odds ratio (aOR), 95 %CI: 1.81, 1.23–2.66), lower perceived financial status (0.57, 0.38–0.83), or parental occupation in non-healthcare (0.38, 0.17–0.89) by multiple logistic regression analysis.

## 4 Discussion

In this study, we present the attitudes toward and knowledge of genetic testing among students from 13 colleges/universities in China's economic powerhouse, Guangdong Province. While our participants exhibited high awareness and positive attitudes toward genetic testing, their functional genetic literacy was only moderate. Our findings raise ethical concerns such as informed consent, privacy, and data security relevant to the genetic testing industry that needs due attention in China.

### 4.1 High awareness and positive attitudes toward genetic testing

Recently, genetic testing has become widely accepted as a prophylactic healthcare option globally. Acceptance rates up to 90 % have been reported among people of diverse demographics in a multicounty study (Chapman et al., 2019; Likhanov et al., 2023). Chinese college students in this provincewide study also had high awareness and overwhelming positivity toward the uptake of genetic tests. For example, more than 90 % of them expressed willingness to undergo at least one type of genetic test, and 65 % were open to DTC home genetic testing, with their predilection for premarital and predictive testing. In contrast, only about 60 % of 492 students from 41 Indonesian universities agreed to genetic screening (Rujito et al., 2020).

Our Chinese students` high awareness and acceptance of genetic testing could be attributed to the active promotion of genetics research and biotechnology as part of the national development strategy by the Chinese government (Mirae Asset Asia Pacific Research, 2020) and increasing commercial campaigns and media coverage about genetic testing (Du and Wang, 2020).

### 4.2 Inadequate functional health literacy

General knowledge about genetics and genetic testing is required for consumers to realize “the right person has the right test at the right time” (Counselors, 2017)—the core concept of making informed choices with genetic testing. Despite the rising global genetic testing trend, the consumers of genetic testing generally do not have adequate knowledge.

Notably, using the International Genetic Literacy and Attitudes Survey, one large study with 4311 participants from 86 countries (the majority from Russia, Nigeria, the USA, and the UK) with diverse demographic and cultural backgrounds and mostly with secondary education, has reported low levels of basic genetic knowledge among their participants, as reflected by their average score of 60.4 %, (Chapman et al., 2019). Slightly lower knowledge levels have been reported in Asian countries. In a study with 867 participants (20–30 years old) from Japan, a country with high literacy rates, the average genetic/genomic knowledge score was 50.9 % (Du and Wang, 2020). Inadequacy in genetic literacy (with an average score of <50 %) was also observed in more than 75 % of Indonesian university students from a nationwide survey (Rujito et al., 2020). Consistent with those studies, our participants exhibited only a moderate level (an average score of 58.8 %) in the functional genetic literacy test. Therefore, it is unlikely that, regardless of their backgrounds, the consumers of genetic testing could make informed choices.

### 4.3 Factors associated with knowledge and positive attitudes toward genetic testing

Some factors are recognized to be associated with the knowledge or attitudes concerning genetics and genetic testing, which include gender (Eum et al., 2018), personality trait (Güler et al., 2022), education (Chapman et al., 2019; Miyoshi and Watanabe, 2023), the medical profession (Miyoshi and Watanabe, 2023), a family or personal history of a genetic disorder (Miyoshi and Watanabe, 2023), religiosity (Pivetti and Melotti, 2013), and financial status (Wang et al., 2021).

In this study, higher functional genetic literacy was independently associated with the female gender, medical student status, health-esteem personality trait, lower religiosity, and self-perceived low financial status. The observed gender difference—with female students demonstrating greater genetic literacy—may reflect their higher academic engagement and intrinsic motivation in health sciences, as previously documented (Hyde and Linn, 2006). Also, medical students' exposure to genetics education likely contributed to their elevated literacy levels. Participants with health-esteem (a self-respect-driven attitude toward healthy living) tend to exhibit proactive knowledge-seeking behaviors. However, as noted in prior research (Baumeister et al., 2003), the relationship between health-esteem and knowledge acquisition is likely reciprocal, where each reinforces the other. The inverse relationship between religiosity and genetic testing knowledge may arise because when the perception of risk is low, conflicts between scientific and religious perspectives become less obvious for less religious people (White, 2009), leading to greater openness to genetic information. The finding of the association between lower perceived financial status and higher literacy is unprecedented. The awareness of genetic testing cost—rather than actual financial status—could have influenced students` perceptions, which warrants future investigation.

For the attitudes toward genetic testing, regression analyses identified self-respect personality as an independent positive predictor, and lower financial status and parental occupation in non-healthcare as independent negative predictors. In the previous multicounty study, the strongest predictors of positive attitudes were genetic knowledge and deterministic beliefs that are difficult or impossible to change (Likhanov et al., 2023). Whereas, in a Romanian study with 1627 adults, self-perceived morality was the strongest predictor of positive attitudes toward genetic testing (Maftei and Danila, 2022).

The association between the attitude and knowledge of genetic testing can be positive, negative, or neutral (LePoire et al., 2019) and the direction of associations (i.e., positive or negative relationships), is inconsistent across the studies, which suggests an involvement of factors other than mere knowledge behind their attitudes and decision-making. Therefore, formulating knowledge and attitude-based interventions could be difficult.

### 4.4 Inadequate informed consent

Informed consent is ethically and legally required before any medical intervention, including genetic testing (Shah et al., 2022). The consumers of genetic testing should have an adequate understanding of the nature of the tests, available alternatives, test accuracy and limitations, interpretation of test results, and potential impacts of test results on the medical, psychological, and social functioning of an individual and family (Matalon et al., 2023).

This study raises concerns regarding the informed decision-making of Chinese college students. For example, 61.6 % of them expressed willingness to get tested even for unpreventable or untreatable conditions, likely due to their curiosity and probably without understanding psychological consequences and ethical challenges, as discussed previously (Maftei and Danila, 2022; Reilly, 2016).

While 72.3 % of our students would decide for themselves whether to have a genetic test or not, nearly all of them (92.9 %) considered that their family or a doctor should be involved in their decision-making (Supplementary File 4). Given the deficient relevant knowledge among the general public as well as the physicians globally and in China (Chen et al., 2022; Counselors, 2017), professional genetic counselors should play a significant role in helping consumers, patients, and families with pre- and post-test counseling. Nonetheless, genetic counseling is not yet a formal profession in China. Only limited counseling services by quasi-genetic counselors are available in some large cities like Shanghai (Sun et al., 2019). This situation can complicate the implementation of public education and counseling interventions.

### 4.5 Genetic testing industry and regulatory gap

Genetic testing service providers are required to offer pertinent information and transparency in their services, such as privacy, data security, and data sharing (Du and Wang, 2020; Matalon et al., 2023), but almost no service providers in China meet those requirements, partly due to the lack of qualified personnel and mainly because of limited regulatory guidance and oversight (Chen et al., 2022; Yichao et al., 2023). For example, a recent review has argued that the Chinese DTC-Genetic testing industry operates as a self-regulated mechanism, with some representative examples as the non-existence of informed consent forms in 94 % and privacy policies in 55.4 % of 83 service providers (Du and Wang, 2020).

This is particularly concerning because our participants considered domestic genetic testing companies the most trustworthy providers. Besides, 73.2 % of them supposedly trust genetic testing companies for privacy and ethical use of their genetic materials. This phenomenon is not surprising as Chinese college students do not have sufficient online health information literacy to identify misinformation and disinformation, and lack cybersecurity literacy (i.e., knowledge, skills, and attitudes toward recognizing cybersecurity risk) (Zhang et al., 2021).

Considering these shortcomings and limitations, people with limited genetic literacy, including Chinese college students in this study, will likely fail to make informed decisions and succumb to complications.

### 4.6 Study strengths and limitations

Our study participants were from 13 colleges in Guangdong province; thus, the study findings should reflect the current situation of future consumers of genetic testing services in China. As our study might have attracted preferentially to those interested in genetic testing, there is a risk of participation bias leading to the observed high rate of positive attitudes. As all available genetic tests in China were listed with their descriptions in the survey, students could have stated their preferred genetic tests by randomly picking from the list without utterly understanding all the medical terms.

### 4.7 Future outlook and suggestions

An increasing trend of congenital birth defects (Zhao et al., 2023) and the introduction of the second-child policy in 2015 and the third-child policy in 2021 will have a considerable impact on the birth rate and the birth prevalence of genetic disorders in China. Most (89.5 %) of our respondents opined genetic testing as their responsibility to pass down healthy genes to future generations. Having such a sense of responsibility suggests a high stake they have in the future of genetic testing. While awaiting a legally and ethically mature genetic testing industry in China, the following practical interventions can be applied:

At the general public level, as suggested in previous studies, target public education on healthy conception (about diet, sleep, exercise, and exposure to harmful substances) (Zhao et al., 2023), stigmatizing people with genetic defects (Matalon et al., 2023), and pre-and post-test genetic counseling (Sun et al., 2019).

At the college level, genetic testing concepts, including risk interpretation and ethical considerations, can be integrated into the medical curricula. Case-based learning, with an emphasis on interpreting genetic test results and making informed decisions in public health contexts, could enhance students' understanding and benefit their future patients. Undergraduate research, such as student-led surveys on genetic literacy-related topics, could also be an option to promote functional genetic literacy among medical and non-medical students. Establishing a campus genetic counseling service operated by volunteer medical students under the supervision of clinical geneticists would further support genetic education and outreach.

At the national level, implementing genetic counseling policies consistent with the current landscape of genomic testing in China, launching genetic counseling training and certification programs across the country, and promoting genetic counseling as a promising professional career in medical and allied health institutions are suggested for China. Although there are many established relevant guidelines and policies in different countries, adopting or adapting the recently established Hong Kong Genetic Counseling Consortium (Tse et al., 2025) would be a practical option.

## 5 Conclusions

Most Guangdong college students exhibited positive attitudes toward genetic testing, preferentially for predictive and premarital genetic tests. With limited relevant literacy, they risk failure to make informed decisions. To mitigate ethical, legal, and social implications, strengthening public education and genetic counseling profession, and regulatory oversight of the Chinese genetic testing industry is strongly suggested.

## CRediT authorship contribution statement

**Jiaming Wang:** Writing – original draft, Visualization, Validation, Investigation, Formal analysis, Conceptualization. **Ruoru Lin:** Validation, Investigation, Formal analysis. **Sijing Luo:** Writing – original draft, Investigation, Formal analysis. **Beilei Zhong:** Validation, Methodology, Investigation. **Yurang Zhu:** Validation, Methodology, Investigation. **Jiayi Huang:** Validation, Methodology, Investigation. **Dangui Zhang:** Writing – review & editing, Visualization, Supervision, Formal analysis. **William Ba-Thein:** Writing – review & editing, Visualization, Supervision, Project administration, Funding acquisition, Data curation, Conceptualization.

## Fundings

This work was supported by the Undergraduate Research Training Program (UGRTP), the 10.13039/100007421Li Ka Shing Foundation (Grant no. LE0003) and 2024 Shantou Science and Technology Plan Medical and Health Project (Grant no. 146). The funding bodies had no role in the design of the study and collection, analysis, and interpretation of data and in writing the manuscript.

## Declaration of competing interest

The authors declare that they have no known competing financial interests or personal relationships that could have appeared to influence the work reported in this paper.

## Data Availability

Data will be made available on request.

## Appendix A Supplementary data

Supplementary material 1. Survey questionnaire Supplementary material 1

Supplementary material 2. Questions for functional genetic knowledge Supplementary material 2

Supplementary material 3. Participants` positive attitudes toward genetic testing and associated factors Supplementary material 3

Supplementary material 4. Awareness and knowledge about genetic testing Supplementary material 4
