# Supplementary material for: Genetic testing and Guangdong college students in China: A cross-sectional study of knowledge and attitudes
Source: Prev Med Rep. 2025 Jun 8;56:103133. doi: 10.1016/j.pmedr.2025.103133 (PMC12205341; doi:10.1016/j.pmedr.2025.103133)
Supplement: Supplementary file 1 — Supplementary material 1. Survey questionnaire [file mmc1.docx]

**Supplementary File 1. Survey questionnaire**

**Part 1: Demographics**

| 1 | Gender/Orientation:   1. Male 2. Female 3. LGBTQ+ |
| --- | --- |
| 2 | Year of birth (e.g., 2001): |
| 3 | Name of university: |
| 4 | Name of college: |
| 5 | Major: |
| 6 | Program:   1. Undergraduate 2. Postgraduate |
| 7 | Personality   1. Health conscious 2. Health esteem 3. Self-respect 4. Perfectionist 5. Worrisome 6. Depression-prone 7. Out-going 8. Shy 9. Others (Please specify): |
| 8 | Current residence (province/city): |
| 9 | Religiosity (How religious are you?)   1. Very much 2. Much 3. Neutral 4. Little 5. Not at all |
| 10 | Parental occupation in healthcare (doctor, nurse, allied health):   1. Yes, please specify: 2. No |
| 11 | Family financial status:   1. Very high 2. High 3. Fair 4. Poor 5. Very poor |

**Part 2: General knowledge about genetics**

|  | | True | False | Don’t know |
| --- | --- | --- | --- | --- |
| 12 | Our genes determine our entire life, including physical and mental well-being. |  |  |  |
| 13 | Some diseases are caused by genes. |  |  |  |
| 14 | Altered (mutated) genes can cause disease. |  |  |  |
| 15 | A person with an altered (mutated) gene may be completely healthy. |  |  |  |
| 16 | Genetic disorders always run in families. |  |  |  |
| 17 | Healthy parents can have a child with an inherited disease. |  |  |  |
| 18 | The child of a person with an inherited disease will always have the same disease. |  |  |  |
| 19 | A genetic test can tell you if you have a higher chance of developing a specific disease. |  |  |  |
| 20 | Genetic testing can detect all genetic disorders. |  |  |  |
| 21 | All genetic disorders can be avoided or prevented if known early. |  |  |  |

**Part 3: Awareness and knowledge about genetic testing**

| Have you heard about | |
| --- | --- |
| 22 | Premarital genetic testing (before marriage)?   1. Yes 2. No |
| 23 | Preconception genetic testing (before getting pregnant)?   1. Yes 2. No |
| 24 | Prenatal genetic testing (after getting pregnant, before giving birth)?   1. Yes 2. No |
| 25 | What could be the benefits of genetic testing to you?   1. Predict the risk of illness 2. Help the patient find the cause 3. Know our own genes 4. Guide fertility 5. Others (Please specify): |

**Part 4: Knowledge about the risks and limitations of genetic testing**

|  | | True | False | Undecided |
| --- | --- | --- | --- | --- |
| 26 | Genetic testing can help you prevent all unwanted consequences from bad genes. |  |  |  |
| 27 | Genetic testing can provide useful information when planning for your future children. |  |  |  |
| 28 | Genetic testing, regardless of the results, can affect you and your family members negatively. |  |  |  |
| 29 | A negative genetic test result ensures that you will not develop a disorder. |  |  |  |
| 30 | A positive genetic test result establishes the risk of developing a disorder. |  |  |  |
| 31 | Genetic test results can be used to predict the course or severity of a condition. |  |  |  |
| 32 | Direct-to-consumer (DTC) home genetic testing kits are good for privacy protection. |  |  |  |

**Part 5: Attitudes towards genetic testing**

|  | | Yes | No | Undecided |
| --- | --- | --- | --- | --- |
| 33 | Would you take predictive genetic tests to know if you are at risk of developing diseases? |  |  |  |
| 34 | If YES to 33, what test would you take for yourself?   1. Whole genome testing 2. Diagnostic testing 3. Predictive and pre-symptomatic genetic tests 4. Carrier testing 5. Pharmacogenomic testing 6. Genetic tumor detection 7. Others (Please specify) |  |  |  |
| 35 | Would you accept direct-to-consumer (DTC) home genetic testing, if available? |  |  |  |
| 36 | Would you get tested for disorders that are still not treatable or preventable? |  |  |  |
| 37 | Would you make decisions by yourself whether to have genetic testing or not? |  |  |  |
| 38 | Would you consult your family and/or doctor before making decisions? |  |  |  |
| 39 | Do you think family members and doctors should be included in decision-making for genetic testing? |  |  |  |
| 40 | Would you take premarital and preconception genetic tests if feasible? |  |  |  |
| 41 | Would you ask your partner to take those genetic tests? |  |  |  |
| 42 | If YES to 40 or 41, what tests would you take (would you ask your partner to take)?   1. Screening of carriers of monogenic hereditary diseases 2. Genetic test for Thalassemia 3. Preimplantation chromosome anomaly detection 4. HPV genotyping (for women) 5. Genetic tests for folic acid utilization (for women) 6. Chromosome Analysis, Peripheral Blood 7. Genetic screening for deafness 8. Others (Please specify) |  |  |  |
| 43 | Would you take (would you ask your partner to take) prenatal genetic tests for your child? |  |  |  |
| 44 | If YES to 43, what tests would you take?   1. Antenatal blood group serology tests 2. Fetal chromosomal aneuploidy 3. DNA non-invasive prenatal genetic testing 4. Folic acid utilization ability gene detection 5. Human bile acid spectrum detection 6. Amniotic fluid metabolite detection 7. Nutritional metabolism gene detection during pregnancy 8. Whole-exome gene detection 9. Others (Please specify) |  |  |  |
| 45 | Would you consider pregnancy termination if your unborn baby has a serious genetic disease? |  |  |  |
| 46 | Would you have your newborn genetically tested to know if s/he is at risk of disease? |  |  |  |
| 47 | If YES to 46, what test would you like your baby tested for?   1. Congenital adrenal hyperplasia detection 2. Lysosome storage disease detection 3. Newborns/children genetic testing (common genetic diseases and safe medication guidance) 4. Deaf gene detection 5. Genetic metabolic disease detection 6. Single gene genetic disease gene detection 7. Others (Please specify) |  |  |  |

**Part 6: Opinions about genetic testing**

| 48 | Do you consider genetic testing a personal responsibility to maintain your own health and to pass down healthy genes to future generations?  a. Yes  b. No |
| --- | --- |
| 49 | Regarding trust in genetic testing providers, which of the following do you consider the most trustworthy?   1. Domestic genetic testing 2. Foreign genetic testing 3. Online genetic testing 4. Others (Please specify) |
| 50 | Do you trust genetic testing companies in protecting your privacy and not misusing your genetic data?   1. Yes 2. No |
