# Supplementary material for: Genetic testing and Guangdong college students in China: A cross-sectional study of knowledge and attitudes
Source: Prev Med Rep. 2025 Jun 8;56:103133. doi: 10.1016/j.pmedr.2025.103133 (PMC12205341; doi:10.1016/j.pmedr.2025.103133)
Supplement: Supplementary file 2 — Supplementary material 2. Questions for functional genetic knowledge [file mmc2.docx]

**Supplementary File 2. Questions for functional genetic knowledge**

| Question item (correct answer ^1^) | Score |
| --- | --- |
| 1. Our genes determine our entire life including physical and mental well-being. (F) | 1 |
| 1. Some diseases are caused by genes. (T) | 1 |
| 1. Altered (mutated) genes can cause disease. (T) | 1 |
| 1. A person with an altered (mutated) gene may be completely healthy. (T) | 1 |
| 1. Genetic disorders always run in families. (F) | 1 |
| 1. Healthy parents can have a child with an inherited disease. (T) | 1 |
| 1. The child of a person with an inherited disease will always have the same disease. (F) | 1 |
| 1. All genetic disorders can be avoided or prevented if known early. (F) | 1 |
| 1. A genetic test can tell you if you have a higher chance of developing a specific disease. (T) | 1 |
| 1. Genetic testing can detect all genetic disorders. (F) | 1 |
| 1. Genetic testing can help you prevent all unwanted consequences from bad genes. (F) | 1 |
| 1. Genetic testing can provide useful information when planning for your future children. (T) | 1 |
| 1. Genetic testing, regardless of the results, can affect you and your family members negatively. (T) | 1 |
| 1. A negative genetic test result ensures that you will not develop a disorder. (F) | 1 |
| 1. A positive genetic test result establishes the risk of developing a disorder. (F) | 1 |
| 1. Genetic test results can be used to predict the course or severity of a condition. (F) | 1 |
| 1. Direct-to-consumer (DTC) home genetic testing kits are good for privacy protection. (F) | 1 |
| Total | 17 |

^1^ Correct answers taken from [1-3]

**References**

1. Martins MF, Murry LT, Telford L, Moriarty F: Direct-to-consumer genetic testing: an updated systematic review of healthcare professionals' knowledge and views, and ethical and legal concerns. *Eur J Hum Genet* 2022, 30(12):1331-1343.

2. Fitzgerald-Butt SM, Bodine A, Fry KM, Ash J, Zaidi AN, Garg V, Gerhardt CA, McBride KL: Measuring genetic knowledge: a brief survey instrument for adolescents and adults. *Clinical genetics* 2016, 89(2):235-243.

3. Chokoshvili D, Belmans C, Poncelet R, Sanders S, Vaes D, Vears D, Janssens S, Huys I, Borry P: Public Views on Genetics and Genetic Testing: A Survey of the General Public in Belgium. *Genetic testing and molecular biomarkers* 2017, 21(3):195-201.
