# Supplementary material for: Genetic testing and Guangdong college students in China: A cross-sectional study of knowledge and attitudes
Source: Prev Med Rep. 2025 Jun 8;56:103133. doi: 10.1016/j.pmedr.2025.103133 (PMC12205341; doi:10.1016/j.pmedr.2025.103133)
Supplement: Supplementary file 3 — Supplementary material 3. Participants` positive attitudes toward genetic testing and associated factors [file mmc3.docx]

**Supplementary File 3. Participants` positive attitudes toward genetic testing and associated factors**

| Genetic test | | Total | Gender | Major | Personality | | | Religiosity | | | Family income | | |
| --- | --- | --- | --- | --- | --- | --- | --- | --- | --- | --- | --- | --- | --- |
|  |  |  | Male | Medicine | Health esteem | Health-conscious | Self-respect | High | Neutral | Low | High | Average | Low |
|  |  | N = 1543 | n = 711 | n = 732 | n = 1179 | n = 1182 | n = 741 | n = 201 | n = 411 | n = 931 | n = 160 | n = 1202 | n = 181 |
| Predictive genetic testing (YES) | | 1286 (83.3) | *P* = 0.632 | *P* = 0.006 | *P* = 0.574 | *P* = 0.294 | *P* = 0.02 | *P* = 0.02 | | | *P* = 0.001 | | |
|  |  |  | 589 (82.8) | 590 (80.6) | 986 (83.6) | 992 (83.9) | 635 (85.7) | 181 (90.0) | 335 (81.5) | 770 (82.7) | 148 (92.5) | 998 (83.0) | 140 (77.3) |
|  | Would you take a genetic profiling test to know if you are at risk of developing diseases? | 1067 (69.2) | *P* = 0.685 | *P* < 0.001 | *P* = 0.541 | *P* = 0.011 | *P* = 0.003 | *P* < 0.001 | | | *P* < 0.001 | | |
|  |  |  | 488 (68.6) | 470 (64.2) | 820 (69.6) | 837 (70.8) | 539 (72.7) | 163 (81.1) | 277 (67.4) | 627 (67.3) | 138 (86.3) | 820 (68.2) | 109 (60.2) |
|  | Would you accept direct-to-consumer (DTC) home genetic testing if available? | 1005 (65.1) | *P* = 0.007 | *P* < 0.001 | *P* = 0.373 | *P* = 0.098 | *P* < 0.001 | *P* < 0.001 | | | *P* < 0.001 | | |
|  |  |  | 438 (61.6) | 427 (58.3) | 775 (65.7) | 783 (66.2) | 527 (71.1) | 158 (78.6) | 274 (66.7) | 573 (61.5) | 134 (83.8) | 764 (63.6) | 107 (59.1) |
|  | Would you get tested for disorders that are still not treatable or preventable? | 951 (61.6) | *P* = 0.514 | *P* < 0.001 | *P* = 0.345 | *P* = 0.24 | *P* = 0.001 | *P* < 0.001 | | | *P* < 0.001 | | |
|  |  |  | 432 (60.8) | 397 (54.2) | 719 (61.0) | 738 (62.4) | 487 (65.7) | 153 (76.1) | 261 (63.5) | 537 (57.7) | 130 (81.3) | 729 (60.6) | 92 (50.8) |
| Premarital and preconception genetic testing (YES) | | 1179 (76.4) | *P* = 0.004 | *P* < 0.001 | *P* = 0.438 | *P* = 0.436 | *P* < 0.001 | *P* = 0.005 | | | *P* < 0.001 | | |
|  |  |  | 519 (73.0) | 527 (72.0) | 895 (75.9) | 909 (76.9) | 598 (80.7) | 171 (85.1) | 316 (76.9) | 692 (74.3) | 140 (87.5) | 914 (76.0) | 125 (69.1) |
|  | Would you take genetic tests if available? | 1117 (72.4) | *P* = 0.001 | *P* = 0.041 | *P* = 0.946 | *P* = 0.02 | *P* < 0.001 | *P* = 0.475 | | | *P* = 0.037 | | |
|  |  |  | 486 (68.4) | 512 (69.9) | 854 (72.4) | 873 (73.9) | 568 (76.7) | 144 (71.6) | 307 (74.7) | 666 (71.5) | 126 (78.8) | 871 (72.5) | 120 (66.3) |
|  | Would you ask your partner to take a genetic test? | 1027 (66.6) | *P* < 0.001 | *P* < 0.001 | *P* = 0.267 | *P* = 0.291 | *P* < 0.001 | *P* < 0.001 | | | *P* < 0.001 | | |
|  |  |  | 437 (61.5) | 433 (59.2) | 776 (65.8) | 795 (67.3) | 529 (71.4) | 161 (80.1) | 276 (67.2) | 590 (63.4) | 133 (83.1) | 797 (66.3) | 97 (53.6) |
| Prenatal genetic testing (YES) | | 991 (64.2) | *P* = 0.036 | *P* < 0.001 | *P* = 0.470 | *P* = 0.137 | *P* < 0.001 | *P* < 0.001 | | | *P* < 0.001 | | |
|  | Would you (or would you ask your partner to) take genetic tests for your child? |  | 437 (61.5) | 427 (58.3) | 763 (64.7) | 771 (65.2) | 515 (69.5) | 160 (79.6) | 263 (64.0) | 568 (61.0) | 127 (79.4) | 771 (64.1) | 93 (51.4) |
| Neonatal genetic testing (YES) | | 909 (58.9) | *P* = 0.459 | *P* < 0.001 | *P* = 0.664 | *P* = 0.349 | *P* < 0.001 | *P* < 0.001 | | | *P* < 0.001 | | |
|  | Would you have your newborn baby genetically tested to know if s/he is at risk of disease? |  | 426 (59.9) | 383 (52.3) | 691 (58.6) | 704 (59.6) | 478 (64.5) | 148 (73.6) | 243 (59.1) | 518 (55.6) | 117 (73.1) | 702 (58.4) | 90 (49.7) |
| Overall positive attitude toward genetic testing ^1^ | | 1410 (91.4) | *P* = 0.173 | *P* = 0.65 | *P* = 0.109 | *P* = 0.107 | *P* = 0.001 | *P* = 0.126 | | | *P* = 0.002 | | |
|  |  |  | 642 (90.3) | 666 (91.0) | 1085 (92.0) | 1088 (92.0) | 696 (93.9) | 191 (95.0) | 371 (90.3) | 848 (91.1) | 153 (95.6) | 1103 (91.8) | 154 (85.1) |

Data shown as n (%) and analyzed by the Chi-square test; ^1^ Those who would consider taking at least one of the above genetic tests.
