# Supplementary material for: Genetic testing and Guangdong college students in China: A cross-sectional study of knowledge and attitudes
Source: Prev Med Rep. 2025 Jun 8;56:103133. doi: 10.1016/j.pmedr.2025.103133 (PMC12205341; doi:10.1016/j.pmedr.2025.103133)
Supplement: Supplementary file 4 — Supplementary material 4. Awareness and knowledge about genetic testing [file mmc4.docx]

**Supplementary File 4. Awareness and knowledge about genetic testing**

| Questions | No. of answering “Yes” (%) |
| --- | --- |
| Have you heard about Premarital genetic test (before marriage)? | 1416 (91.8) |
| Have you heard about Prenatal genetic test (before getting pregnant)? | 1358 (88.0) |
| Have you heard about Preconception genetic test (after getting pregnant, before giving birth)? | 1402 (90.9) |
| Benefits to do genetic testing |  |
| Predict the risk of illness | 1395 (90.4) |
| Help the patient find the cause | 1085 (70.3) |
| Know our own genes | 1273 (82.5) |
| Guide fertility | 1134 (73.5) |
| Others ^1^ | 13 (0.8) |
| Do you consider genetic testing a personal responsibility to maintain your own health and to pass down healthy genes to future generations? | 1381 (89.5) |
| Which of the following genetic testing do you consider the most trustworthy? |  |
| Domestic genetic testing | 1139 (73.8) |
| Foreign genetic testing | 335 (21.7) |
| Online genetic testing | 43 (2.8) |
| Others ^2^ | 26 (1.7) |
| Do you trust genetic testing companies in protecting your privacy and not misusing your genetic data? | 1129 (73.2) |

^1^ included no benefits, adjunctive therapy, and inherited diseases prevention; ^2^ included I don’t know, not sure, not trust any genetic testing, any genetic testing is ok, never heard of genetic testing, both domestic and foreign genetic testing are ok, based on government recommendation, and self-detection.
